# Exploring the Quality of Life (QOL) of medical students in Karachi, Pakistan

**DOI:** 10.1186/s12909-024-05481-4

**Published:** 2024-05-03

**Authors:** Makhdoom Bilawal, Ramna Shafique, Rafay Shahab Ansari, Muhammad Arsalan Bashir, Muhammad Amaan Nadeem, Sardar Noman Qayyum, Hussain Haider Shah, Annoushay Tehseen, Lujain Alnemr, Samim Noori

**Affiliations:** 1 Ziauddin Medical University, Karachi, Pakistan; 2 Indus Hospital & Health Network, Karachi, Pakistan; 3 Bacha Khan Medical College, Mardan, Pakistan; 4 Dow University of Health Sciences, Karachi, Pakistan; 5 Rawal Institute of Health Sciences, Islamabad, Pakistan; 6 University of Health Sciences - Hamidiye International School of Medicine, Istanbul, Turkey; 7 Nangarhar University Faculty of Medicine, Nangarhar, Afghanistan

**Keywords:** Medical education, Dental education, Medical students, Quality of life, Well-being, Burnout

## Abstract

**Background:**

The pursuit of medical and dental education is challenging and can affect the overall quality of life of medical students. Assessing the quality of life of medical students is the first step in the preparation of efficient future health care professionals. This study used the World Health Organization Quality of Life Brief Version (WHOQOL-BREF) to evaluate the quality of life of medical and dental students in Karachi, Pakistan.

**Objectives:**

The study objectives include: assessing the QoL of medical and dental students and their general health satisfaction and self-satisfaction.

**Materials and methods:**

This cross-sectional study was conducted among 344 medical and dental students from different medical and dental schools in Karachi, Pakistan. The World Health Organization Quality of Life Brief Version (WHOQOL-BREF) questionnaire was used to assess QOL, which included 26 items covering four domains: physical, psychological, social, and environmental. All scores for the domains ranged from 4 to 20. Scoring was done according to the WHOQOL-BREF procedure manual. The questionnaire was disseminated to medical students using Google Forms. SPSS software was used to analyze the data. Cronbach’s alpha and the Kaiser-Meyer-Olkin (KMO) test were used to evaluate the reliability and sampling adequacy of the data for factor analysis. Descriptive statistics were computed for each variable and QoL domain, including frequencies, percentages, averages, and standard deviations. Domain scores were compared using a t-test and one-way ANOVA, with p-values less than 0.05, indicating statistical significance.

**Results:**

Among the 344 medical students, 56.7% (*n* = 195) were female and 43.3% (*n* = 149) were male. The WHOQOL-BREF demonstrated excellent reliability, with a Cronbach’s alpha of 0.918. Most medical students rated their overall QOL (62.2%) and health satisfaction (46.8%) as good, and were able to get around well (71.3%). No significant sex differences were found across the various QOL domains. Marital status significantly affected QOL scores (*p* < 0.005). Single students had significantly higher QOL scores than married/separated/divorced students did. Overall, the environmental domain had the highest mean score (26.81 ± 6.17), while social relationships had the lowest mean score (9.68 ± 2.93).

**Conclusion:**

The findings of this study provide valuable insights into the QoL of medical and dental students. Most participants reported moderate satisfaction with their physical health and lower satisfaction with the psychological, social, and environmental components of QoL. Marital status was found to significantly impact the QoL as compared to single students with greater QoL. These findings can help form targeted interventions to enhance medical students’ quality of life and prepare efficient future healthcare professionals.

## Background

The World Health Organization (WHO) defines Quality of Life (QOL) as an individual’s belief about their position in life with regard to culture and principles, including their aims, standards, desires, and worries [1]. The concept of quality of life in the context of culture and value systems is widely accepted. It has drawn the attention of various populations, which is reflected in the growing significance of determining and enhancing general well-being and life satisfaction [2]. The QoL can be evaluated with a wide variety of instruments including the WHOQOL (World Health Organization Quality of life), the EuroQol Group’s EQ-5D, the Short Form health surveys (SF-36 & SF-12) and Q-LES-Q (Quality of life Enjoyment and Satisfaction Questionnaire) [3]. However, the WHOQOL isa well-established and widely used tool to assess quality of life across four domain:. physical health, psychological health, social relationships and environment. It is designed for cross-cultural comparisons and has been translated into more than 40 languages. A condensed version of the WHOQOL-BREF, consisting of 26 items, is considered suitable for use in clinical trials where concise measures are required, and in epidemiological studies where quality of life may be among multiple outcome variables. The WHOQOL-BREF has also been validated in multi-cultural studies [4] and for medical students [5] to assess the quality of life.

Medical and dental education are two distinct fields of professional education, although in Pakistan dental students are considered a part of broader medical education system. In our study, we will use the terms ‘medical education’ and ‘medical students’ for the Bachelor of Medicine and Bachelor of Surgery (MBBS) students and ‘dental education’ and ‘dental students’ for the Bachelor of Dental Surgery (BDS) students. Both medical education and dental education are widely acknowledged as highly challenging and stressful endeavors. The rigorous curricula, demanding workload, and clinical training can place significant strain on the cognitive and learning capabilities of both medical and dental students. Studies [6–13] have consistently demonstrated that both medical and dental students experience elevated levels of stress throughout their undergraduate education. The consistent pressure to acquire and retain vast amounts of complex knowledge, while also developing critical clinical skills, can lead to cognitive overload and burnout among students in both medical and dental disciplines. Furthermore, the emotional toll of dealing with patient care, and medical/dental emergencies can exacerbate the stress experienced by medical and dental students which can interfere with their ability to effectively learn, retain and apply the knowledge and skills.

Research suggests that the commencement of both medical and dental programs is associated with a decline in student’s mental health because they experience higher levels of stress and are at a greater risk of developing depressive symptoms as they embark on their professional education journey [14]. Many students experience higher levels of academic stress because of the strict and competitive environment that promotes competition rather than teamwork. Despite mental health challenges, students rarely seek assistance for their issues [15]. 

Medical/dental education and training can adversely affect the physical and mental wellbeing of students [8]. Medical and dental education is often lengthy accompanied by academic stress with limited employment prospects, demanding course work, and extensive study hours. As a result, some medical students struggle to maintain better grades throughout the medical school. Therefore, medical students exhibit higher susceptibility to stress, anxiety, burnout, and depression than non-medical students and the general population [7–9].

Studies in other countries [10–13] also reported the similar findings as well. The study conducted by F Youssef in Trinidad and Tobago [11] revealed that 40% of medical students experienced depression, while 52% reported burnout. Chang et al.’s study [10] conducted among medical students of United States also revealed burn out in 55% of medical students and depressive symptoms in 60% of the medical students. A study among dental students in Mexica [12] reported emotional exhaustion and higher levels of stress perception in 52% and 42.3% respectively. Another study revealed significant association of burnout syndrome with poor performance, medication intake due to depressive symptoms and intention to drop out among Brazilian dental students [13]. These studies highlight the widespread occurrence of mental-health related challenges among both medical and dental students worldwide.

The quality of life of medical and dental students is crucial as it impacts their academic success, professional development, patient care and personal well-being. Addressing the challenges faced by these students supports the cultivation of resilient, empathetic and high-performing healthcare providers for the future. A study [16] comparing the quality-of-life medical students with non-medical younger population revealed that more than half of the medical students had worsened psychological well-being and social relationships with one-quarter having lower physical and environmental scores as well. There were no significant variations across various academic years but female students had worse physical and psychological well-being as compared to male students. Another study also reported lower QOL scores among female students and students with chronic comorbidities [17].

In Pakistan, medical education faces a range of complex challenges as the public demand for better-trained healthcare professionals is increasing. Young doctors are requesting to improve facilities and provide more training opportunities, specialists are seeking programs to continue education, and both students and teachers in medical colleges are dissatisfied with existing regulatory system [18]. 

In the past few years significant development has taken place in medical education in Pakistan including the introduction of newer examinations i.e. National Licensing Exams (NLE) and the replacement of the Pakistan Medical and Dental Commission (PMDC) with the Pakistan Medical Commission (PMC) in October 2019. However, after three years of implementation, these amendments were reversed, and the status of PMDC was restored along with the cancellation of NLE. At present, the PMDC regulates medical education activities in Pakistan and does not mention the quality of life of medical students in the “PM&DC Medical and Dental Education Policy and Regulations 2023’ [19]. Initially, there were concerns of the World Federation for Medical Education (WFME) regarding the recognition of PMDC [20], but the recognition was granted in February 2024.

Financial issues as well as other challenges faced by medical students such as psychosocial factors further impact their well-being and quality of life [21]. Hence, there is a need for QOL evaluation of medical students in Pakistan after changes in policies and economic downturn in the country to enable relevant authorities to intervene to improve students QoL.

Previous studies on the QoL of medical students in Pakistan have utilized the WHOQOL-BREF questionnaire [22–24]. However, due to recent financial and educational crises, significant differences are anticipated in the QOL findings with those of previous studies. Therefore, it is hypothesized that PMDC policy revision has significantly impacted the quality of life medical and dental students in Karachi, Pakistan as measured by the WHOQOL-BREF instrument. These circumstances are likely to negatively impact the well-being and life satisfaction of medical students, necessitating fresh evaluation of their QoL.

The objectives of this study were (a) to assess the QOL of medical and dental students across four domains: physical, psychological, social relationships, and environmental, and (b) to assess overall QOL, general health satisfaction, and self-satisfaction among medical and dental students in Karachi, Pakistan.

## Methods

### Study design

A descriptive cross-sectional study was conducted among 344 medical and dental students from private and public medical universities of Karachi, Pakistan. Institutional review board approval was obtained from the Ziauddin University to comply with all ethical regulations that may apply to the study. The ethical guidelines outlined in the Declaration of Helsinki were followed in this study.

### Study population

The study population comprised medical and dental students enrolled in private and public medical universities in Karachi, Pakistan, between the ages of 18 and 25. The total medical and dental students enrolled in various medical and dental colleges of Karachi between the age of 18 and 25 years were approximately 3450 with Karachi having the largest enrollments of medical students in Pakistan. The following medical colleges participated in this study: Ziauddin Medical University, Dow Medical College (DMC), Dow International Medical College (DIMC), Jinnah Sindh Medical University (JSMU), United Medical and Dental College (UMDC), Jinnah Medical and Dental College (JMDC), Karachi Medical and Dental College (KMDC), Liaquat College of Medicine and Dentistry, and Liaquat National Hospital and Medical College.

### Study sample

The study sample size of 344 was calculated using open-source software openepi.com sample size calculator with 95% confidence interval, 5% margin of error.

### Inclusion/exclusion criteria

An age range of 18 to 25 years and enrollment in a medical and dental school were eligible to participate in the study. Non-medical students and students under the age of 18 or over the age of 25 years were considered ineligible.

### Data collection and tool

Data were collected using the online Google Forms questionnaire. Online questionnaires were disseminated to medical students using WhatsApp. Consent was obtained prior to answering the questionnaire. The English version of the WHOQOL-BREF was used to evaluate the QoL of medical students. It contains 26 items over four domains of QoL as identified by the WHO: physical health, psychological health, social relationships, and environmental. 5-ponts Likert scale scoring was scored on a five-point Likert scale, with 1 indicating the lowest score and 5 indicating the highest score. The sum of scores from all four domains indicates quality of life, with higher scores indicating a better quality of life.

### Statistical analysis

Statistical analyses were performed using SPSS version 24. It was completed in three phases. Although the WHOQOL-BREF instrument used has already been validated in previous studies, the internal consistency and reliability of the WHOQOL-BREF on a specific population of medical students has not been confirmed. Cronbach’s alpha and Kaiser-Meyer-Olkin (KMO) tests were used to assess the reliability and sampling adequacy for factor analysis. Descriptive statistics, such as frequencies, percentages, means, and standard deviations, were calculated for all variables and domains of QoL, in which the scores of each domain were transformed into a linear scale ranging from 0 to 100 and then expressed as means and standard deviations of the total scores. Finally, the QoL scores for each domain were compared by applying a t-test and one-way analysis of variance (ANOVA), with p-values less than 0.05, which were used to compare domain scores.

## Result

### Reliability & validity

The English version of the WHOQOL-BREF [25] demonstrated good overall internal consistency and reliability, with a Cronbach’s alpha coefficient of 0.918. Cronbach’s alpha for all four domains was also calculated, and the questionnaire demonstrated good reliability for each domain with a coefficient value greater than 0.7 is regarded as acceptable. Cronbach’s alpha was 0.841 for the physical health domain, 0.760 for the psychological domain, 0.856 for the social relationship domain, and 0.894 for the environmental domain.

The KMO test was used to evaluate sampling adequacy. The test result was 0.948, indicating that the data were suitable for the factor analysis. Bartlett’s Test of Sphericity further supported the data for factor analysis and yielded a chi-square value of 6567.758 with a significant p-value (*p* < 0.001) and standard deviation of 465.

### Demographic details

Among the 344 participants in our study, 56.7% (*n* = 195) were females and 43.3% (*n* = 149) were males. The majority (41%, *n* = 141) of participants were aged between 22 and 23 years, followed by 18–19 years age group (28.8%, *n* = 99), 24–25 years age group (20.6%, *n* = 71), and 20–21 years age group (9.6%, *n* = 33). Of the 344 participants, 329 (95.2%) were single, 11 (3.2%) were married, three (0.9%) were separated, and one (0.2%) was divorced.

### Comparison of QOL scores across all four domains

The environmental domain had the highest mean score (26.81 ± 6.17), followed by physical health with a mean score of 22.13 ± 3.82, psychological health with a mean score of 20.11 ± 4.05, and social relationships with a mean score of 9.68 ± 2.93. When the mean scores were compared based on sex and age, no significant differences were found across the four domains. However, the comparison based on marital status showed significant differences, with single students having higher scores than married/separated/divorced students across all four QoL domains (*p* < 0.005). Table 1 shows a comparison of the various domains of quality of life.

**Table 1:** Comparison of quality of life domains. (Table Legend: This table compares the scores of students in various domains of quality of life, such as physical health, psychological health, social relationships, and environment, analyzed using demographic variables, such as gender, age, and marital status. For each variable, a summary of the responses is provided with p-values from statistical tests comparing domain scores between demographic groups. This table provides insights into significant differences in QoL scores based on gender, age group, and marital status with lower p-values (*p* < 0.05), indicating stronger evidence)

Variables	*n* (%)	Physical health		Psychological health		Social relationship		Environment	
		Mean ± SD	*p*-value	Mean ± SD	*p*-value	Mean ± SD	*p*-value	Mean ± SD	*p*-value
Gender									
Male	149 (43.3%)	22.30 ± 3.82	0.477	20.18 ± 3.81	0.778	9.75 ± 3.04	0.702	27.06 ± 6.13	0.528
Female	195 (56.7%)	22.00 ± 3.83	0.477	20.05 ± 4.24	0.778	9.63 ± 2.86	0.702	26.63 ± 6.21	0.528
Age									
18–19	99 (28.8%)	22.0 ± 3.94	0.153	19.86 ± 3.98	0.283	9.48 ± 2.97	0.103	26.57 ± 5.80	0.097
20–21	33 (9.6%)	22.51 ± 3.92	0.153	19.51 ± 4.99	0.283	9.96 ± 2.86	0.103	26.78 ± 6.26	0.097
22–23	141 (41%)	22.54 ± 3.58	0.153	20.60 ± 3.70	0.283	10.07 ± 2.69	0.103	27.68 ± 5.60	0.097
24–25	71 (20.6%)	21.32 ± 4.04	0.153	19.73 ± 4.32	0.283	9.08 ± 3.28	0.103	25.46 ± 7.38	0.097
Marital status									
Single	329 (95.2%)	22.29 ± 3.78	0.002	20.34 ± 3.88	< 0.005	9.82 ± 2.84	< 0.005	27.21 ± 5.90	< 0.005
Married	11 (3.2%)	19.36 ± 3.44	0.002	16.00 ± 5.23	< 0.005	7.45 ± 3.64	< 0.005	19.81 ± 6.11	< 0.005
Separated	3 (0.9%)	16.66 ± 1.52	0.002	12.33 ± 0.57	< 0.005	4.33 ± 0.57	< 0.005	13.66 ± 2.51	< 0.005
Divorced	1 (0.2%)	17.0 ± 00.00	0.002	13.00 ± 00.0	< 0.005	4.00 ± 00.0	< 0.005	14.00 ± 0.0	< 0.005

### Overall quality of life and health satisfaction

**Fig. 1:**
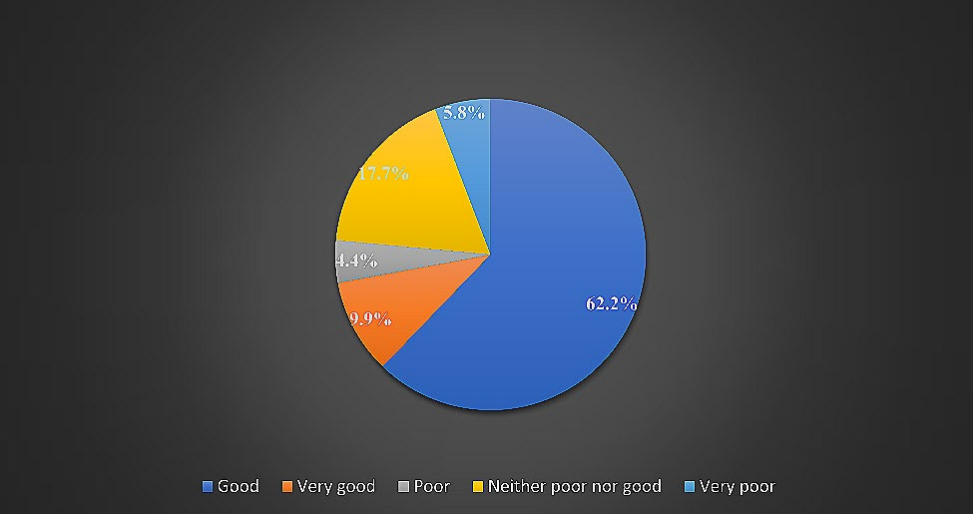
Student assessment of quality-of-life satisfaction. (According to the WHOQOL-BREF instrument, the majority of students (*n* = 212, 62.2%) reported a good quality of life, 61 (17.7%) reported neither poor nor good, 34 (9.9%) reported very good, 20 (5.8%) reported very poor, and 15 (4.4%) reported poor.)

**Fig. 2:**
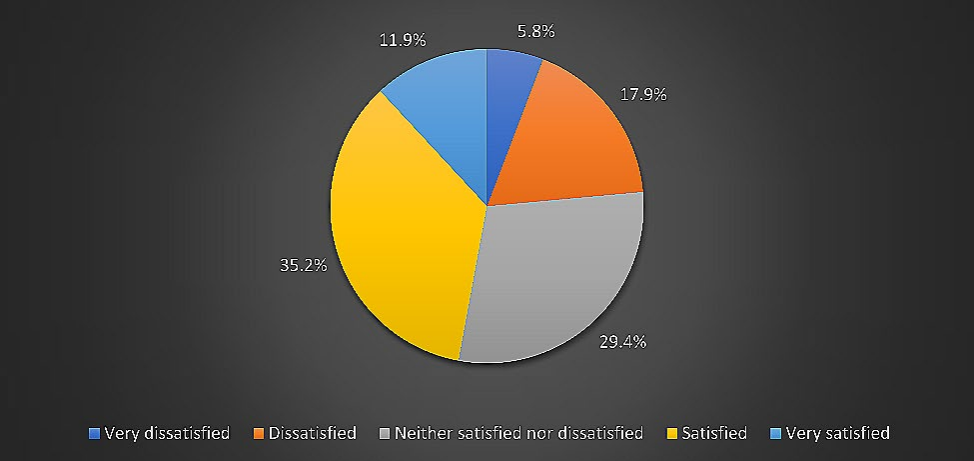
Levels of self-satisfaction among students. (According to the WHOQOL-BREF, 41 (11.9%) students were very satisfied, 20 (5.8%) were very dissatisfied, and 101 (29.4%) were neither satisfied nor dissatisfied. The majority of students (35.2%, *n* = 121) were satisfied, while 61 (17.9%) were dissatisfied with themselves.)

As shown in Fig. 1, most students (*n* = 214, 62.2%) rated their overall quality of life as good, while 4.4% (*n* = 15) rated it as poor. Of the 344 medical students, 35.2% (*n* = 121) were satisfied with themselves, while 5.8% (*n* = 20) were very dissatisfied, as depicted in Fig. 2.

**Fig. 3:**
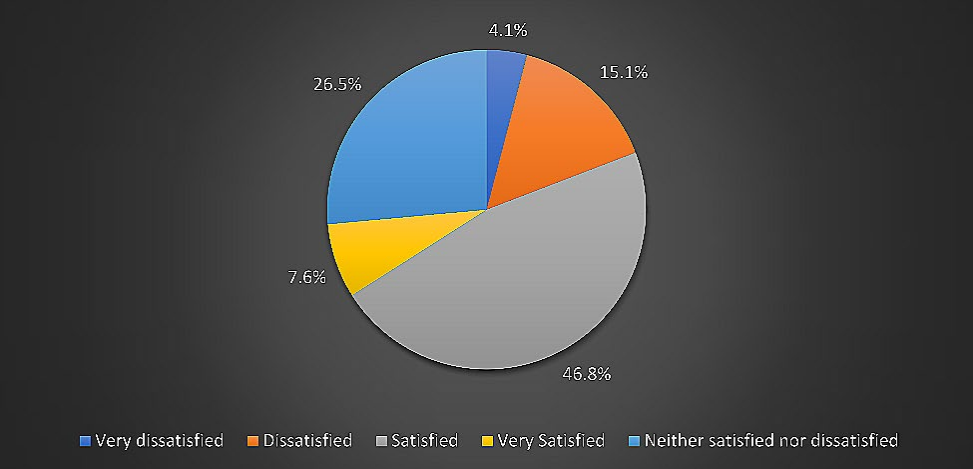
Survey of student’s satisfaction regarding their health. (According to the WHOQOL-BREF instrument, about 161 students (46.8%) were satisfied with their health, 91 students (26.5%) were neither satisfied nor dissatisfied, 52 (15.2%) were dissatisfied, 26 (7.6%) were very satisfied, and only 14 (4.1%) were very dissatisfied with their health.)

Regarding health satisfaction, most students (46.8%, *n* = 161) reported being satisfied, 7.6% (*n* = 26) reported being very satisfied (Fig. 3), and only 4.1% (*n* = 14) were very dissatisfied with their current health status.

### Mobility and physical health

Most students (71.3%) reported being able to get around well. However, half of the medical students reported frequent or regular physical pain, which restricted their daily activities. Nearly half of the medical students reported the need for regular medical treatment. (Table 2)

### Mental and emotional health

Being able to concentrate was rated highly by most students, with 45% reporting good and 5.8% reporting very good concentration ability. One-third of students frequently experienced negative emotions related to anxiety and emotion, highlighting mental health issues. Sleep quality is impertinent to both physical and mental health; dissatisfaction with sleep quality was also common, with only 42.4% reporting satisfaction to some extent. (Table 2)

### Detailed assessment of QOL

As shown in Table 2. Students were asked to rate specific aspects over the past two weeks; a majority of the students reported enjoying life (46.5%), feeling their life was meaningful (37.8%), being able to concentrate well (39.2%), and feeling safe (43.3%). Most students (33.7%) reported that their physical environments were neither healthy nor unhealthy. Dissatisfaction with personal relationships and sex life was found in approximately one-third of the students. Table 2 summarizes the detailed QOL assessment of QOL.

**Table 2:** Self-reported quality of life. (Table Legend: Table 2 summarizes the responses of the 344 students to the WHOQOL-BREF questionnaire. Each respondent could choose one of the five options, indicating frequency, agreement with the statement, or their level of satisfaction. The options are listed in columns, from the most negative on the left to the most positive on the right. This table also contains the number and percentage of respondents who selected each option)

Questions	Very Poor	Poor	Neutral	Good	Very Good
1. How would you rate your quality of life?	20 (5.8%)	15 (4.4%)	61 (17.4%)	214 (62.2%)	34 (9.9%)
2. How well are you able to get around physically?	16 (4.7%)	30 (8.7%)	53 (15.4%)	198 (57.6%)	47 (13.7%)
***Questions***	***Not at all***	***A little***	***Moderately***	***Very much***	***Extremely***
3. To what extent do you feel that physical pain prevents you from doing what you need to do?	25 (7.3%)	45 (13.1%)	105 (30.5%)	147 (42.7%)	22 (6.4%)
4. How much do you need any medical treatment to function in your daily life?	52 (15.1%)	52 (15.1%)	79 (23.0%)	125 (36.3%)	36 (10.5%)
5. How much do you enjoy life?	11 (3.2%)	37 (10.8%)	95 (27.6%)	160 (46.5%)	41 (11.9%)
6. To what extent do you feel your life to be meaningful?	12 (3.5%)	37 (10.8%)	106 (30.8%)	130 (37.8%)	59 (17.2%)
7. How well are you able to concentrate?	15 (4.4%)	48 (14.0%)	126 (36.6%)	135 (39.2%)	20 (5.8%)
8. How safe do you feel in your daily life?	21 (6.1%)	30 (8.7%)	109 (31.7%)	149 (43.3%)	35 (10.2%)
9. How healthy is your physical environment?	14 (4.1%)	52 (15.1%)	116 (33.7%	140 (40.7%)	22 (6.4%)
10. Are you able to accept your bodily appearance?	24 (7.0%)	40 (11.6%)	124 (36.0%)	120 (34.9%)	36 (10.5%)
11. Have you enough money to meet your needs?	15 (4.4%)	46 (13.4%)	97 (28.2%)	149 (43.3%)	37 (10.8%)
12. Do you have enough energy for everyday life?	18 (5.4%)	48 (14.0%)	108 (31.4%)	147 (42.7%)	23 (6.7%)
13. How available to you is the information that you need in your day-to-day life?	15 (4.4%)	37 (10.8%)	113 (32.8%)	142 (41.3%)	37 (10.8%)
14. To what extent do you have the opportunities for leisure activities?	22 (6.4%)	50 (14.5%)	106 (30.8%)	142 (41.3%)	24 (7%)
***Question***	***Never***	***Seldom***	***Quite often***	***Very often***	***Always***
15. How often do you have negative feelings such as blue mood, despair, anxiety, depression?	72 (20.9%)	56 (16.3%)	90 (26.2%)	105 (30.5%)	21 (6.1%)
***Questions***	***Very dissatisfied***	***Dissatisfied***	***Neutral***	***Satisfied***	***Very satisfied***
16. How satisfied are you with your sleep?	33 (9.6%)	79 (23.0%)	86 (25.0%)	118 (34.3%)	28 (8.1%)
17. How satisfied are you with your ability to perform your daily living activities?	16 (4.7%)	61 (17.7%)	120 (34.9%)	122 (35.5%)	25 (7.3%)
18. How satisfied are you with your capacity for work?	24 (7.0%)	66 (19.2%)	95 (27.6%)	124 (36.0%)	35 (10.2%)
19. How satisfied are you with yourself?	20 (5.8%)	61 (17.7%)	101 (29.4%)	121 (35.2%)	41 (11.9%)
20. How satisfied are you with your personal relationships?	25 (7.3%)	59 (17.2%)	100 (29.1%)	116 (33.7%)	44 (12.8%)
21. How satisfied are you with your sex life?	36 (10.5%)	53 (15.4%)	122 (35.5%)	96 (27.9%)	37 (10.8%)
22. How satisfied are you with the support you get from your friends?	24 (7.0%)	57 (16.6%)	103 (29.9%)	118 (34.3%)	42 (12.2%)
23. How satisfied are you with the conditions of your living place?	15 (4.4%)	56 (16.3%)	100 (29.1%)	116 (33.7%)	57 (16.6%)
24. How satisfied are you with your access to health services?	19 (5.5%)	64 (18.6%)	95 (27.6%)	129 (37.5%)	37 (10.8%)
25. How satisfied are you with your transport?	22 (6.4%)	64 (18.6%)	112 (32.6%)	102 (29.7%)	44 (12.8%)
26. How satisfied are you with your health?	14 (4.06%)	52 (15.1%)	91 (26.5%)	161 (46.8%)	26 (7.6%)

## Discussion

The objective of this study was to determine the quality of life (QOL) of medical students in Karachi. The findings suggest that WHOQOL-BREF is a reliable tool to evaluate the quality of life of medical students in Karachi. The WHOQOL-BREF examines the satisfaction of respondents across significant life domains, therefore these measurements are profoundly impacted by “the cultural context and value systems” prevalent within the population [26]. Our study found that the environmental domain mean scores were the highest followed by physical health domain. The psychological health domain followed by social relationship domain had the least domain scores. Previous studies conducted among medical students in Saudi Arabia [22] and Pakistan [27] also had the highest environmental domain mean scores (67.81 and 70.43 respectively) as compared to other domains. Unlike our study, the physical health domain had the least mean score in the Saudi Arabian study and psychological domain had the least score in another Pakistani study. The different outcomes of these studies can be attributed to several factors including the relatively stable political and environment in Saudi Arabia. Unlike Pakistan, the relative stability in Saudi Arabia may positively influence the psychological well-being and social relationships of the medical students. In contrast, Pakistan’s less stable and supportive environment may lead potentially leading to greater challenges for students in maintaining psychological well-being and social relationships.

In our study, we found no significant association of QoL domains’ mean scores with age and gender groups, despite some studies found significant gender differences [17, 28]. Previously studies have demonstrated significant gender differences (*p* < 0.05) across various domains of QoL, with males exhibiting higher scores in the physical health domain [17, 29] and psychological health domain [28, 30] as compared to females. However, our study’s findings indicate that despite the potential societal and cultural norms that may impose variations in the lived experiences of males and females in Pakistani society, there appears to be no significant difference in the overall QoL between the two genders. It may also point to the effectiveness of societal and institutional efforts to address and mitigate gender-based inequalities, resulting in a more balanced and inclusive QoL perception among medical and dental students.

Due to the demanding nature of medical and dental education, it can impair the development of social relationships [20, 21] which is consistent with the findings of our study showing only 33.4% satisfied with their personal relationships and social relationships domain with least mean score among all four QoL domains. High levels of stress and academic pressure are generally associated with depression, anxiety, and burnouts in students globally [31]. Inam et al. [32] assessed the anxiety and depression levels among medical students of a private university Pakistan and found that 60% of medical students had anxiety and depression. Similarly, in our study most of the medical students (62.8%) frequently reported negative emotions associated with anxiety and depression. Hence there is the need for counselling and mental health support services for students.

In our study, marital status significantly impacted the QOL scores (*p* < 0.05). Single students had higher scores than married/separated/divorced students across all four domains of QoL. Han et al.’s study [33] found the similar findings with singles having better QoL than married under the age of 30. Unlike our study findings, a study conducted among Saudi Arabian dental students [34] reported that married dental students had higher QoL scores across all domains but also had higher satisfaction with QoL and general health as compared to single students. This outcome is suggestive of social maturity, partner’s support and companionship as a result of marriage. However, this difference in outcomes in these studies can be explained in economical and cultural contexts.

The limitations of this study include: (a) The sample size was small, being drawn from a single city. (b) The study did not analyze medical and dental students as separate groups, preventing comparison of QOL domain scores between these two student populations. (c) The study also didn’t include the comparison of QOL domain scores of various academic years, though the existing literature suggests that QoL domain scores increase from first year to third year followed by a decline in QOL scores from third year to fifth year [28]. The findings suggest that interventions should be implemented during the third year to address the observed decline in QOL among students. This targeted approach could help mitigate the drop in QOL. d)The study did not collect sociodemographic information, such as financial conditions, that could have been used to correlate with QOL domain scores across different groups. e) The study also lacked any socio-cultural information that could have been used to investigate how cultural variations may have impacted the QOL domain scores.

A further nationwide follow-up study will be conducted to gain a more comprehensive understanding of the quality of life among medical and dental students in Pakistan. This upcoming study will take into account the financial and cultural background of the participants, providing valuable insights on potential predictors of QoL of Pakistani population of medical and dental students. Longitudinal studies should also be devised to assess fluctuations in QoL over a period of time.

The study provides valuable insights into the QoL of medical and dental students in Karachi, Pakistan, particularly in the context of the recent economic collapse and policy shifts. The study findings highlight the importance of providing psychological support and guidance to these students on maintaining a balance between their social relationships and professional obligations. The study revealed that higher levels of stress and academic pressure which is inherent in the healthcare education significantly impede the students’ ability to preserve both their psychological well-being and social connections. These findings emphasize the need for targeted interventions, such as support programs that promote mental health, foster healthy social relationships, and encourage balanced lifestyles among future healthcare professionals. By informing the development of such tailored initiatives, the study’s findings can help optimize the overall quality of life for medical and dental students in Pakistan.

## Conclusion

This cross-sectional study provides valuable insights into the QoL of medical and dental students in Karachi, Pakistan. The findings indicate that most medical students rated their overall QoL as ‘moderate’. Most medical students also reported high satisfaction with physical health but low satisfaction with psychological, social and environmental domains. Most students reported frequently experiencing negative emotions associated with anxiety and depression. Our study found no significant sex differences in QoL domain scores but marital status had significantly impacted WoL scores. Single students had better QoL domain scores as compared to married/separated/divorced students. However, our study provides valuable insights on QoL of medical and dental students in Karachi, Pakistan after economic collapse and policies shifts regarding medical and dental education. The study findings indicate that with economic downturn and changes in policies regarding the medical and dental education has significantly impacted students’ psychological wellbeing and social relationships domains of QoL especially these conditions have affected students with relationships status married/separated/divorced. The findings can help form targeted interventions such as support programs promoting mental well-being, social relationships and healthy lifestyles to optimize medical and dental students QoL.

## Data Availability

The data that support the findings of this study are available from the corresponding author, Samim Noori, upon reasonable request.
